# Idiopathic Epiretinal Membrane: Microvasculature Analysis with Optical Coherence Tomography and Optical Coherence Tomography Angiography

**DOI:** 10.3390/tomography8010016

**Published:** 2022-01-12

**Authors:** Klaudia Ulfik-Dembska, Sławomir Teper, Michał Dembski, Anna Nowińska, Edward Wylęgała

**Affiliations:** 1 Clinical Department of Ophthalmology, Faculty of Medical Sciences in Zabrze, Medical University of Silesia, Poniatowskiego 15, 40-566 Katowice, Poland; slawomir.teper@gmail.com (S.T.); michau32@gmail.com (M.D.); anna.nowinska@sum.edu.pl (A.N.); rekroz@sum.edu.pl (E.W.); 2 District Railway Hospital, Panewnicka 65, 40-760 Katowice, Poland

**Keywords:** idiopathic epiretinal membrane, optical coherence tomography, optical coherence tomography angiography, macular microvasculature, retinal thickness, foveal avascular zone

## Abstract

Background: The present study examined the relationships among macular microvasculature, retinal structure, and epiretinal membrane (ERM) and explored the utility of optical coherence tomography (OCT) angiography (OCTA) in idiopathic ERM assessment. Methods: The study sample comprised 276 eyes of 276 patients. A total of 154 eyes with ERM and 122 normal (control) eyes were analyzed. Only one eye of each participant was randomly selected for posterior segment imaging. Each patient underwent OCT and OCTA. Images were analyzed with AngioTool 0.6. Results: Foveal avascular zone was significantly smaller in the ERM group (*p* = 0.044). Average retinal thickness and foveal thickness were significantly higher in the ERM group (both *p* = 0.001). Moreover, 64 (41.5%) patients exhibited no metamorphopsia, while 46 (29.8%) and 44 (28.7%) patients exhibited moderate and extensive metamorphopsias, respectively. Meanwhile, FAZ was negatively correlated with central retinal thickness in the ERM group. The vessel area (*p* = 0.0017) and vessel percentage area (*p* = 0.044) were significantly greater in the ERM group. Conclusions: Changes observed in the superficial plexus in OCTA are related to the severity of metamorphopsia and can be further evaluated to support decision making regarding the surgical management of idiopathic ERM.

## 1. Introduction

Epiretinal membranes (ERM) are a fibrocellular contractile proliferation that form over the surface of the internal limiting membrane of the retina, usually in the macular area. They were first described by Iwanoffin in 1865. Epiretinal membranes can be idiopathic or secondary to other ocular pathologies, such as retinal detachment, uveitis, retinal vascular occlusions, and trauma [1]. Importantly, idiopathic ERM formation does not depend on complete PVD; rather, it often begins during early stages of PVD when one or more vitreomacular adhesions remain [2]. Epiretinal membranes are proliferations at the vitreoretinal junction, after posterior vitreous detachment [3]. Increased prevalence of ERM is noted in the population older than 60 years, and peak prevalence is observed in those between 70 years and 79 years (11.63%). Additionally, because the ERM was found to be associated with PVD in 70% of patients at the time of the diagnosis, PVD has been considered as an important pathogenic factor in ERM formation. The treatment depends on the patient’s symptoms (such as metamorphopsia) and visual loss, and it consists of vitrectomy with membrane peeling, usually aided by coloring agents [4]. Although ERM is typically detected through fundus examination, the use of optical coherence tomography (OCT) has increased detection sensitivity [5]. Since its invention in the 1990s, OCT has been widely applied for the diagnosis and evaluation of retinal diseases [6]. Subsequently, optical coherence tomography angiography (OCTA) was developed as a new detection method for retinal and choroidal diseases. OCTA enables visualization of the vascular network and blood flow using an algorithm known as split-spectrum amplitude-decorrelation angiography [7,8]. OCTA has been applied for the detection of retinal and choroidal diseases, such as aged-related macular degeneration (AMD), choroidal neovascularization (CNV), diabetic retinopathy, and chorioretinopathy [9]. However, there are only few reports on the use of OCTA for the assessment of retinal vascularization in patients with ERM. It is difficult to qualify a patient with ERM for surgery. The symptoms are highly subjective, and the deterioration of visual acuity is often slight. In this light, more objective morphological factors indicating the severity of the disease must be identified. These factors must be directly related to retinal function and symptom severity. Although the retinal thickness in the macula is one of these factors, it may not be sufficiently reliable, as relatively substantial macular edema in ERM may not necessarily deteriorate the vision markedly. ERM leads to changes in retinal thickness and morphology, as well as the vascular network, which is evident in fundus examination.

The aim of the present study was to examine the relationships among macular microvasculature, retinal structure, and ERM and to explore the utility of OCTA for ERM assessment. Another purpose of the study was determine the association between changes in the vascular network and severity of metamorphopsia.

## 2. Materials and Methods

### 2.1. Study Participants

This controlled, prospective, observational study was approved by the Ethics Committee of the Medical University of Silesia, Katowice, Poland (KNW/0022/KB1/86/18) and was conducted in accordance with the ethical standards stated in the 1964 Declaration of Helsinki. All patients had to sign an informed consent form before any study procedure, and then patients were qualified for the research project. The participants were informed of the nature, purpose, and method of research. A total of 154 eyes of patients with epiretinal membrane and 122 eyes in the control group were analyzed.

The inclusion criteria were presence of idiopathic ERM, consent to participate in the study, and age >18 years. The exclusion criteria were as follows: high myopia or diabetes, history of uveitis, pars plana vitrectomy, penetrating eye injuries, obstacles in obtaining sufficient pupil dilation, media opacities resulting in low-quality imaging, and pregnancy.

### 2.2. Examination Procedure

For all patients, we collected detailed clinical history. The patients underwent ophthalmologic examination using a slit lamp (SL 990 Digital Version; CSO; Firenze, Italy), measurement of the best corrected visual acuity (BCVA), tonometry (Goldmann applanation tonometry), optical coherence tomography, and optical coherence tomography angiography. We measured BCVA using the Snellen visual acuity charts. Data on previous and current ophthalmic history, age, lens status, and sexwere collected. Each patient with ERM was examined for the presence of metamorphopsia according to the scale from 0 to 2 (0, none; 1, moderate; 2, extensive), as reported upon self-assessment and confirmed on the basis of the presence of distortions in the Amsler grid test.

**Figure 1:**
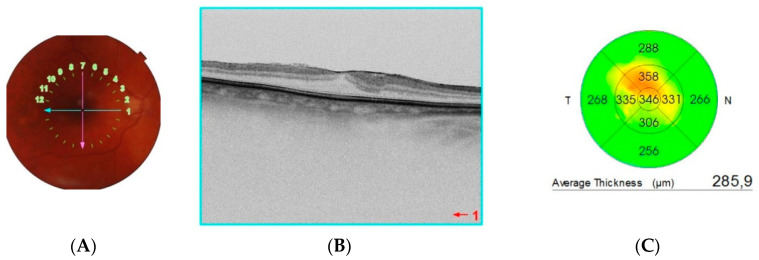
(**A**) Fundus photograph; (**B**) optical coherence tomography scan showing the epiretinal membrane traction causing foveal distortion; (**C**) retina thickness map.

**Figure 2:**
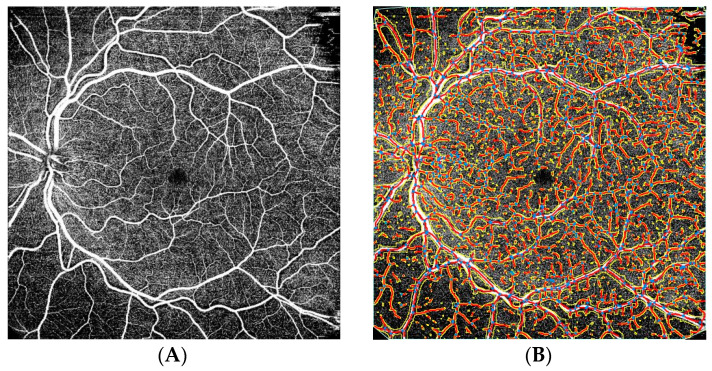
Scans of the macula and peripapillary region in a patient with epiretinal membrane (**A**) using optical coherence angiography; (**B**) vessel analysis using AngioTool.

All patients underwent OCT (SS-OCT, Triton, Topcon Healthcare, Tokyo, Japan) (6 × 6 mm) (1024 × 12) and 12 × 12 mm scans) and OCTA (12 × 12 mm scans) of the radial macula Figure 1. Scans of the macula and peripapillary region were obtained by the same experienced investigator. Eye tracking and a projection artefact removal algorithm were used to provide high transverse and axial resolution. OCTA images were analyzed with AngioTool 0.6 (National Cancer Institute) (Figure 2). AngioToolis used for the quantitative analysis of angiogenesis and various morphometric and spatial parameters of the vessels, including vessel length and density, branching index, and lacunarity. AngioTool is open-source software that uses a fast, multi scale, Hessian-based filter and a fast, recursive Gaussian filter for the enhancement of the vessel structure. Only the superficial plexus was analyzed due to the high number of artefacts and low analytical reliability in the deep capillary plexus.

### 2.3. Statistical Analysis

Statistica version 13.1 (TIBCO Software Inc., Palo Alto, CA, USA) with Penguinand Pandas statistical packages for Python was using to perform statistical analysis. A *p*-valueless than 0.05 was considered significant, considering multiple testing for interpretation. Quantitative variables were analyzed by calculating the mean, standard deviation, median, minimum, and maximum. We used the Shapiro–Wilk test to examine the normality of the variable distribution. Statistical significance was calculated using the *t*-test for parametric variables and Mann–Whitney U-test for nonparametric variables. Nonparametric Spearman’s correlation coefficients were used to determine statistical dependence. The Kruskal–Wallis test was used to confirm the significance of differences.

## 3. Results

In our study, a total of 154 eyes with ERM and 122 normal (control) eyes were analyzed. We randomly selected for posterior segment imaging only one eye of each participant. The mean age of patients in the ERM group was 71.41 ± 8.19 (range 53–86) years and that of patients in the control group was 59.26 ± 21.21 (range 21–83) years. A total of 68 (55.73%) eyes in the control group were of females and 54 (44.26%) were of males. On the other hand, 112 (72.72%) eyes with ERM were of females and 42 (27.27%) were of males. In the control group, 96 (78.68%) eyes were phakic, and 26 (21.31%) were pseudophakic, whereas 102 (66.23%) eyes were phakic in the ERM group, and 52 (33.76%) were pseudophakic. The mean axial length of the eyeball in the control group was 23.43 mm, while that in the ERM group was 23.51 mm (*p* = 0.34). Patients with high myopia were excluded from the initial stage of the study.

The mean visual acuity was 0.204 logMARin the ERM group and 0.079 logMARin the control group. The mean intraocular pressure was 14.31 ± 2.82 (range 11–21) mmHg in the ERM group and 14.52 ± 2.43 (range 11–19) mmHg in the control group.

Table 1 presents a comparison of the results obtained by OCT in the study groups. In the ERM group, the variables foveal thickness (*p* < 0.001) and variable average macular thickness (*p* < 0.001) were normally distributed (*p* > 0.05, Shapiro–Wilk test). Foveal avascular zone (FAZ) (*p* < 0.001), average macular thickness (*p* < 0.001), and foveal thickness (*p* < 0.001) were significantly different between the two groups.

FAZ was significantly smaller in the ERM group. In contrast, average retinal thickness and foveal thickness were significantly higher in the ERM group (both *p* = 0.001). FAZ and retinal thickness were negatively correlated.

FAZ measured 329.57 ± 171.14 µm in the ERM group and 378.67 ± 171.23 µm in the control group. There were no significant differences in FAZ between females and males (*p* = 0.21, Mann–Whitney U-test). The average retinal nerve fiber layer (RNFL) thickness was comparable between the two study groups (*p* = 0.74). Superior and inferior RNFL thickness was also comparable between the two study groups (*p* = 0.264 and *p* = 0.25, respectively). Mean RNFL thickness in the four quadrants was 96.91 ± 17.29 µm (IC95, 100.95) in the ERM group and 96.09 ± 17.07 µm (IC95, 100.47) in the control group.

The effect of ERM on RNFL thickness was negligible. However, in 21 (13.63%) patients, RNFL thickness had to be measured with an OCT software caliper to correct for artefacts related to ERM in the vicinity of the optic disc. MeanRNFL thickness in the four quadrants was 98.34 ± 14.12 µm without correction and 96.52 ± 17.18 µm with correction (*p* = 0.035).

Table 2 presents a comparison of the results obtained by OCTA and assessed using AngioTool in the study groups. The vessel area (*p* = 0.0017) and vessel percentage area (*p* = 0.044) were significantly greater in the ERM group. The number of junctions was higher in the ERM group (*p* < 0.001). The vessel length was higher in the ERM group (*p* = 0.0002).

The total number of end points was higher in the ERM group. In the ERM group, vessel area (*p* = 0.36), vessel percentage area (*p* = 0.75), total number of junctions (*p* = 0.91), and junction density (*p* = 0.76702) were normally distributed (*p* > 0.05, Shapiro–Wilk test). In the control group, vessel area (*p* = 0.096), vessel percentage area (*p* = 0.86), total number of junctions (*p* = 0.13), junction density (*p* = 0.57), and mean lacunarity (*p* = 0.06) were normally distributed (*p* > 0.05, Shapiro–Wilk test). There were significant differences in the following normally distributed variables between the two groups (*t*-test): vessel area (*p* = 0.0017) and total number of junctions (*p* = 0.000044). There was a significant difference in total vessel length (*p* = 0.0002, Mann–Whitney U-test) between the two groups.

We analyzed the correlation between FAZ and metamorphopsia severity in patients with idiopathic epiretinal membrane.

**Figure 3:**
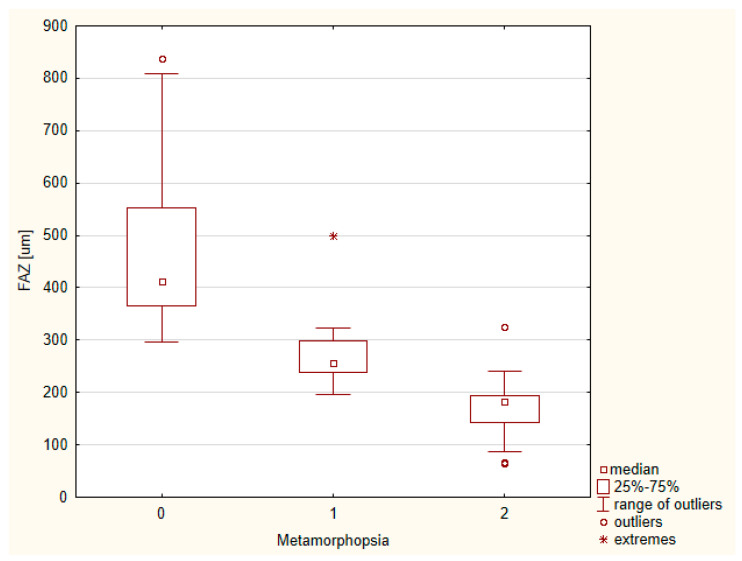
Graph of correlation between foveal avascular zone and metamorphopsia severity.

We performed ANOVA using the Kruskal–Wallis test, which showed the presence of differences with *p* < 0.05. Correlation analysis revealed that a smaller FAZ was related to greater image distortion in patients with ERM. As such, 64 (41.5%) patients exhibited no metamorphopsia, while 46 (29.8%) and 44 (28.7%) patients exhibited moderate and extensive metamorphopsia, respectively (Figure 3). Meanwhile, FAZ was negatively correlated with central retinal thickness in the ERM group. Moreover, ERM significantly affected retinal microvascularization.

**Figure 4:**
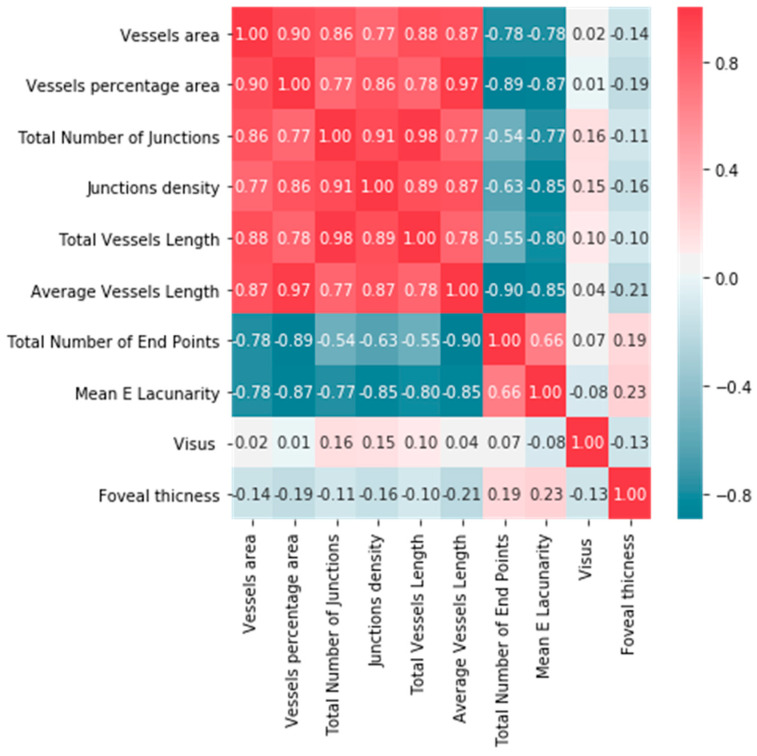
Correlation matrix of parameters obtained in OCT, OCTA, and BCVA.

**Figure 5:**
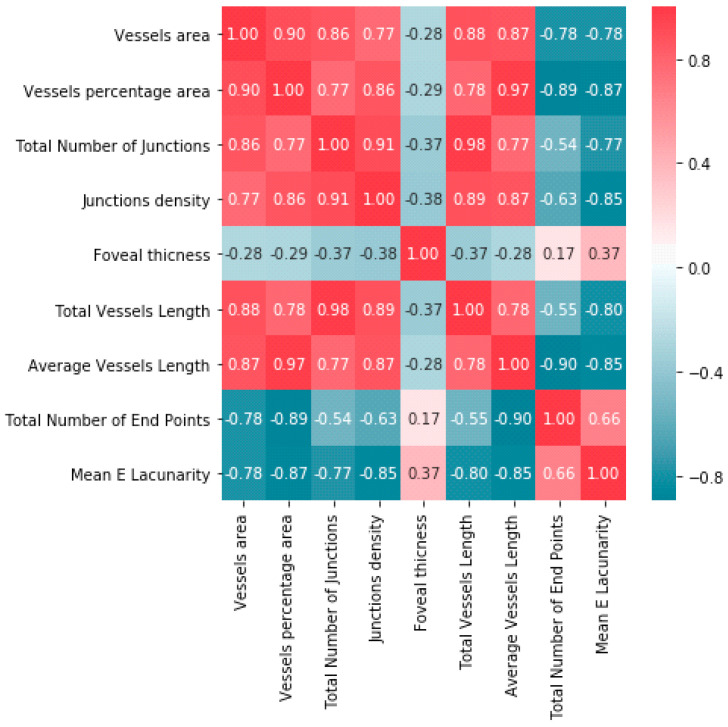
Correlation matrix of parameters obtained in OCT and OCTA.

We analyzed the correlation between parameters obtained in the OCT, OCTA, and BCVA (Figure 4 and Figure 5).

To determine the strength of dependency between the variables, the matrix of correlations was analyzed. Graphs representing Spearman’s correlation coefficients are provided in Figure 4 and Figure 5. The relationships among OCT, OCTA, and BCVA were studied.

We conducted numerous studies of the correlation between individual parameters. Correlation coefficients were calculated. A strong positive correlation was found between vessel area and total number of junctions (0.86), as well as between vessel area and total vessel length (0.88). A strong negative correlation was found between vessel percentage area and total number of end points (0.87), as well as between junction density and lacunarity (0.85). A weak negative correlation was found between central retinal thickness and visual acuity in ERM patients (−0.13). A positive correlation between foveal thickness and the degree of severity of metamorphopsia was found.

**Figure 6:**
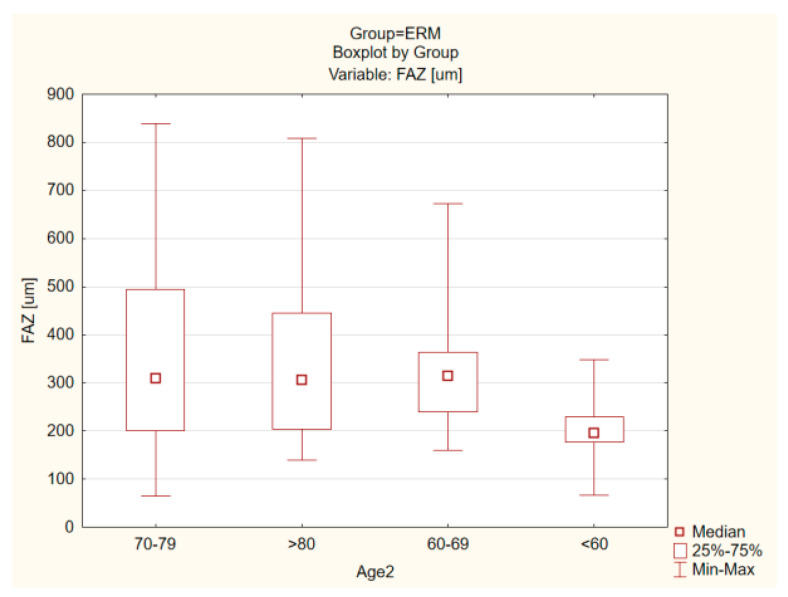
Comparison of age in the ERM group for the FAZ parameter (*p* = 0.724).

**Figure 7:**
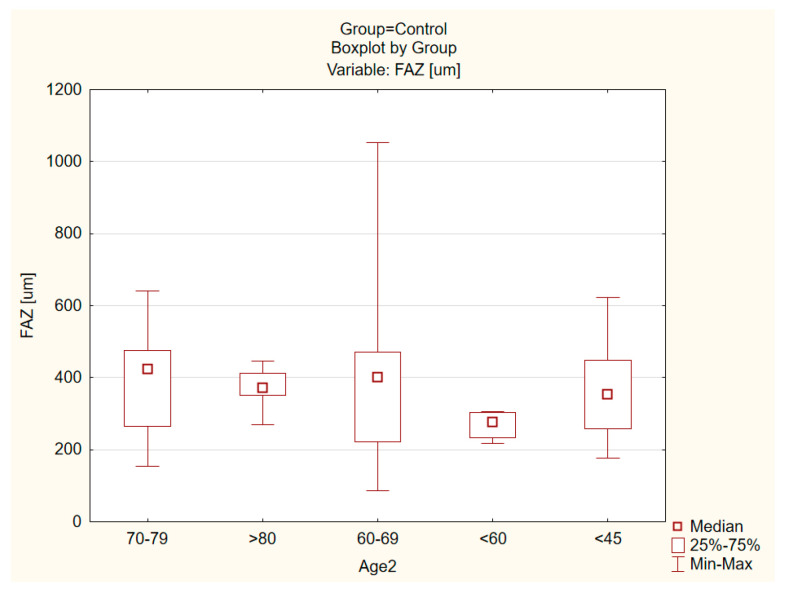
Comparison of age in the control group for the FAZ parameter.

No statistically significant differences according to the Mann–Whitney U-test were found in terms of gender and age for all parameters in both OCT and OCT-A. There were no statistically significant differences with regard to the condition of the lens in the ERM group and the control group (Figure 6 and Figure 7).

In the control group, there was a statistically significant Spearman correlation for the variables age and quality macula (*r* = −0.404); in the ERM group, this correlation is much smaller and insignificant (*r* = −0.04).

There were no differences in the ERM, where as, in the control group, there was a difference for quality macula due to gender (*p* = 0.016).

## 4. Discussion

The present study demonstrated a significant association between ERM and retinal microvasculature changes.

Using a vessel enhancement algorithm, we successfully extracted retinal vasculature and measured the number of retinal vessel branch points, vascular area, and vessel length with AngioTool. AngioTool is frequently utilized to quantitatively assess vessel morphometric and spatial parameters [10,11].

OCTA can be used to evaluate FAZ diameter and vessel density. FAZ is a capillary-free area bordered by the foveal capillaries running in the inner retinal layers. The area of FAZ is correlated with sex, age, and foveal thickness [11]. The effect of ILM peeling on FAZ is well known [12,13,14,15]. In the present study, FAZ area in the ERM group was significantly smaller than that in the control group. Spearman’s correlation analysis showed that a smaller FAZ area was negatively correlated to a thicker fovea in the ERM group. This trend is consistent with the conclusions of previous Japanese studies [13,14,15]. In those previous studies, FAZ area was shown to increase following vitrectomy. In the present study, the mean foveal thickness in the ERM group was significantly higher than that in the control group. Samara et al. [16] described a significant negative correlation between FAZ area and central foveal thickness (CFT) in normal eyes. Our results confirmed the negative correlation between FAZ area and CFT in both control and ERM groups. Similar results have been reported in Japanese and American studies [15,17,18]; additionally, these previous studies found that foveal thickness decreased following vitrectomy. Moreover, foveal thickness was higher in the ERM eyes than in normal eyes, indicating that the tractional effects of ERM might persist even after its removal. The integrity of the ellipsoid zone was significantly correlated to postoperative vision [15]. Additionally, the final effect of the operation depends on changes of the foveal vasculature, which disperses the light before it reaches the photoreceptor cells [18]. Measurement of FAZ area in patients with ERM is paramount, because the degree of metamorphopsia is significantly correlated with the area of FAZ in these patients [19]. The morphology, structure, and area of FAZ are predictive factors for the requirement of retinal membrane surgery [20]. The present study confirmed the association between FAZ area and metamorphopsia severity. Patients with a small FAZ are more likely to suffer from severe metamorphopsia.

AngioTool has been frequently used for the quantitative assessment of vessel morphometric and spatial parameters [11]. The projected OCTA slabs were analyzed with AngioTool, and retinal vasculature and branch point number, vascular area, and vessel length were automatically measured. Foveal vessel density in the ERM group was significantly higher than that in the control group. A study from Switzerland used AngioTool to compare the quality of four OCTA modules [21]. To the best of our knowledge, however, no study has used AngioTool to compare macular vascularity in patients with ERM and healthy individuals. This tool has been used to assess macular vascularity in patients with AMD or CNV before and after treatment [22]. Lacunarity is a measure of vessel non-uniformity, with a high value reflecting in homogeneity and a low value reflecting homogeneity in vascular structure. Normalized blood vessels are characterized by a greater coverage of pericytes and are less leaky, tortuous, and dilated [23]. Mean lacunarity was higher in the control group of this study. The use of AngioTool to objectively and quantitatively evaluate microvascular structure in patients with ERM is an important strength of our study, although image evaluation with thresholding has some limitations. Thresholding results may vary depending on the threshold values or methods. Another issue related to thresholding is the large variation in measurements in images of healthy individuals [24,25]. Some differences in the observed parameters in the ERM group may be related to the greater exposure of the elevated vessels in the superficial capillary plexus in OCTA and may be apparent. Nonetheless, numerical values can be used to compare and differentiate patients for the purposes of research.

There are limitations in this study. First, we included relatively few cases. Second, we analyzed only the superficial capillary plexus to avoid the segmentation errors occurring in eyes with ERM and the current low resolution of deep plexus imaging in OCTA, rendering AngioTool useless.

## 5. Conclusions

Changes observed in the superficial capillary plexus in OCTA, particularly the FAZ area, are associated with these verity of metamorphopsia and may serve as an additional objective parameter indecision making related to vitreoretinal surgery.

## Figures and Tables

**Table 1:** Comparison of the results of the parameter values obtained by optical coherence tomography and OCTA in the studied groups. The * *p*-value was calculated using the Shapiro–Wilk test for each of the parameters.

	ERM Group						Control Group					
Parameter	Mean ± Standard Deviation	IC 95	Median	Minimum	Maximum	*p* *	Mean ± Standard Deviation	IC95	Median	Minimum	Maximum	*p* *
Foveal avascular zone (FAZ) (um)	329. 57 ± 171.14	373.78	302.73	64.39	838.62	0.001	378.67 ± 171.23	426.8387	364.1510	86.5740	1053.428	<0.001
Foveal thickness (um)	265.64 ± 72.96	282.21	260.00	104.00	432.00	0.35	212.08 ± 38.25	221.88	200.00	159.00	325.00	0.0002
Averange macular thickness (um)	282.99 ± 33.62	290.62	281.30	127.90	397.60	0.631	268.94 ± 13.77	272.47	269.90	226.90	307.20	0.12
Average retinal nerve fiber layer (RNFL) (um)	96.91 ± 17.29	100.95	99.00	42.00	128.00	0.0011	96.09 ± 17.07	100.47	98.00	43.00	126.00	0.0005
Superior quadrant RNFL thickness (um)	114.50 ± 28.87	121.24	118.00	40.00	177.00	0.0003	111.09 ± 22.67	116.90	113.00	46.00	148.00	0.007
Inferior quadrant RNFL Thickness (um)	116.35 ± 28.5489	123.01	121.00	23.00	155.00	<0.001	122.75 ± 29.05	130.19	125.00	34.00	193.00	0.006

**Table 2:** Comparison of the results of obtained using optical coherence tomography angiography and AngioTool. The * *p*-value was calculated using the Shapiro–Wilk test for each of the parameters; ** *p* denotes the significance of differences between the control and study groups.

	ERM Group						Control Group						
Parameter	Mean ± Standard Deviation	IC 95	Median	Minimum	Maximum	*p* *	Mean ± Standard Deviation	IC95	Median	Minimum	Maximum	*p* *	*p* **
Vessels area	102,783.3 ± 16,912.49	107,312.5	103,929.0	54,550.0	134,812.0	0.36	84,591.0 ± 26,185.56	91,881.1	89,033.0	29,643.0	127,731.0	0.09	0.0017
Vessels percentage area	40.1 ± 6.50	41.9	40.2	26.6	57.8	0.75	37.8 ± 8.58	40.2	38.8	20.8	59.9	0.86	0.0444
Total number of junctions	1079.0 ± 249.13	1145.8	1074.5	557.0	1722.0	0.91	847.0 ± 315.09	934.7	829.5	288.0	1533.0	0.13	<0.0001
Junctions den sity	0.004 ± 0.001	0.004	0.004	0.002	0.007	0.76	0.004 ± 0.001	0.004	0.004	0.001	0.007	0.57	0.577
Total vessels length	21,547.6 ± 2889.62	22,321.4	21,715.0	10,635.7	27,502.0	0.014	17,974.3 ± 5166.66	19,412.7	19,328.4	7303.5	25,800.3	0.003	0.0002
Total number of end points	1088.1 ± 215.22	1145.7	1100.0	248.0	1472.0	0.0022	959.0 ± 355.29	1057.9	1039.5	209.0	1623.0	0.004	0.074

## Data Availability

The data presented in this study are available in Chair and Clinical Department of Ophthalmology, Faculty of Medical Sciences in Zabrze, Medical University of Silesia in Katowice, 40-055 Katowice, Poland.

## Abbreviations

ERM	Epiretinal membrane
OCT	Optical coherence tomography
OCT-A	Optical coherence tomography angiography
BCVA	Best corrected visual acuity
FAZ	Foveal avascular zone
RNFL	Retinal nerve fiber layer
CFT	Central foveal thickness
ILM	Internal limiting membrane
DR	Diabetic retinopathy
PVD	Posterior vitreal detachment
